# The Libyan doctors' brain drain: an exploratory study

**DOI:** 10.1186/1756-0500-2-242

**Published:** 2009-12-08

**Authors:** Hani TS Benamer, Amin Bredan, Omran Bakoush

**Affiliations:** 1 Department of Neurology, New Cross Hospital, Wolverhampton, UK; 2 Neurosciences Department, University of Birmingham, UK; 3 Department of Biomedical Molecular Biology, Ghent University, 927 Technologiepark, 9052 Ghent, Belgium; 4 Department for Molecular Biomedical Research, Flanders Institute for Biotechnology (VIB), 927 Technologie Park, 9052 Ghent, Belgium; 5 Department of Nephrology, Clinical Sciences, Lund University, Sweden

## Abstract

**Background:**

Medical emigration from developing to developed countries is a well established phenomenon of substantial importance. Though Libya is classified as an upper-middle income country, it has been affected by this trend. This study was undertaken to identify some of the possible reasons behind the emigration of Libyan doctors and factors that might motivate them to return.

**Findings:**

Seventy-four completed questionnaires were analysed. Median age of the respondents was 43 years (33-60) and median duration of stay outside Libya was 15 years (6-29). Most of the participants were resident in Europe (66%). The desire to further their education and research was the main reason given by 88% of the respondents for leaving Libya, while 50% of them gave that as the main reason for staying abroad. One-third of the respondents (31%) cited economic factors as the main reason for not returning. None of the respondents ruled out returning to Libya, and about half of them stated that they definitely or probably will return to Libya. 58% ranked reform of the Libyan health system as the most important reason that could induce them to return to Libya.

**Conclusion:**

The study shows that reforming the health care system in Libya might induce some of the physicians who moved abroad mainly for educational and economic reasons to return to Libya to practice medicine.

## Background

The last few years have witnessed rising interest in analysing various issues related to migration of physicians from developing countries. Recent publications have tried to shed light on the number of migrating physicians [1,2], the reasons for their migration [3-6], the impact of emigration on the source countries [3,4], and what steps can be taken to stem this flow [7].

Libya is a North African Arab country. It has a surface area of 1,775,500 km^2 ^and a population of 5.3 million. One-third of the population is under the age of 15 years, and the majority of the population lives in the coastal cities, the most prominent of which are Tripoli (the capital) and Benghazi (second largest city). Libya had been an Italian colony from 1911 until the end of the Second World War, when it became a British protectorate. In 1951 Libya gained its independence and was established as a kingdom. In 1969 Colonel Muammar Qadhafi took over and he has been leading the country since then.

The discovery of oil in the 1960s completely transformed the socioeconomic status of the country. As living standards rose in the 1970s, the number of medical schools and health service facilities increased. However, in the 1980s Libya's relations with the United States and European countries deteriorated, and its political isolation culminated in United Nation sanctions in the 1990s. This had negative repercussions on the country's infrastructure. However, in recent years Libya has been rapidly re-emerging from international isolation.

The Libyan public health sector is the main provider of health services, which are provided free at the point of delivery. Twenty-seven specialised hospitals and 36 general hospitals in different parts of the country provide secondary and tertiary care. There are 39 polyclinics, 23 communicable disease centres and 1165 primary health care units and centres [8]. Private practice was banned in the early 1980s but has been reinstituted in recent years. Absence of a health insurance system and uncertainty about the position of private investors limit the role of the private health care sector [8]. Over the years, the organisation of the health services was changed from a centrally controlled system to a completely decentralised system and, two years ago, back again to full centralisation. This vacillation led to serious effects on the quality of the health services. Libyan citizens perceive the public health system as inadequate if not poor, and consequently health tourism to neighbouring Tunisia and Egypt has flourished [9].

The first Libyan medical school was established in Benghazi in 1970, followed by the one in Tripoli in 1973. Between 1987 and 2001 the number of medical students increased dramatically as seven new medical schools were established in different parts of the country. The medical education system is based on the traditional British curriculum and uses English as the language of instruction.

Like many other Arab and African countries, Libya has been affected by the drain of its doctors. Clemens and Pettersson reported a total of 585 Libyan physicians when they used the destination-country census data of 2000 to estimate the number of African-born doctors who had immigrated to the United Kingdom (UK), the United States of America (USA), Australia, Canada, France, Spain, and Belgium by [1]. Mullan, when examining physician immigration to UK, USA, Australia and Canada in 2004, reported that 624 Libyan doctors were practicing in these countries and that 63% of them were in the UK [2]. Mullan estimated that 8.9% of all Libyan physicians are practicing in the USA, UK, Canada or Australia [2]. Moreover, Libya was among the top five African countries when Arah calculated physician migration density, i.e. the number of migrating physician per 1000 population [10].

This study is an attempt to identify some of the reasons behind the emigration of Libyan doctors and the factors that might induce them to return. By highlighting the problem, our results might assist in planning and execution of further studies of this important subject in more depth.

## Methods

A simple questionnaire consisting of three parts was designed [Additional File 1]

Part I contains background questions about age, sex, year of departure from Libya, place of primary medical qualification, current country of residence, and type of current job. It also asks about how settled the respondent feels both professionally and socially in his or her current country of residence.

Part II asks about the reasons for initially leaving Libya and the reasons for later deciding to settle abroad. The respondents were given four options: educational (to pursue further education and research), economic (to seek a better income and living standard), personal (family or other personal reasons), and other (the respondents could explain in their own words). Respondents were allowed to select more than one answer, but in such cases they were asked to prioritise their choices as 1, 2, 3, and 4, one being the most important and four being the least important.

Part III asks about the likelihood of returning to Libya to practice medicine. Five options were provided: definitely, probably, possibly, unlikely and never. The respondents were also asked about what would induce them to return to practice medicine in Libya. They were given five options. The first, significant reform of the Libyan health care system, was meant and has been used by Libyan doctors and health officials in a global sense that covers both health care deliveries *per se *and physician employment conditions. The other options were significant improvement in the prospects for research in Libya, social reasons, other reasons (the respondents could explain in their own words), and not returning under any circumstances. The respondents were allowed to choose more than one answer, in which case they were asked to prioritise their choices as 1, 2, 3, and 4, as described above. The respondents were finally asked about the type of employment they would take up if they were to return to Libya: public hospital, academic institution, private sector, non-medical job, or other.

Our target consisted of doctors who were Libyan citizens and practicing abroad. We recruited participants from the Libyan Journal of Medicine database (the only international Libyan medical journal, sponsored mainly by expatriate Libyan doctors) and the Libyan Doctors Society (a society of Libyan doctors abroad). We also asked potential participants to forward us the e-mails of any expatriate Libyan doctors they knew. In this way we obtained 225 e-mail addresses of Libyan doctors practicing outside Libya, and we targeted them all. The purpose of the study was explained, and confidentiality and anonymity were pledged. The responses of the first ten respondents were reviewed carefully and a note was sent to clarify that the question concerning the country of qualification meant the country in which the first medical degree was obtained. A reminder was sent after two weeks to those who had not responded. In total, 78 questionnaires were returned, giving a response rate of 35%. Respondents who had been abroad for five years or less were excluded, which left 74 questionnaires for analysis. We excluded these respondents because many Libyan graduates are funded by the Libyan government during their post-graduate training abroad, which lasts a few years. Hence, they cannot be considered to have settled abroad during that period of sponsorship. The responses of the remaining respondents were entered in Microsoft Excel for descriptive analysis.

## Results

### Background information

Only one of the respondents was a female. Median age of the respondents was 43 years (33-60 years) and median duration of stay outside Libya was 15 years (6-29 years). Most of them were resident in Europe (66%), 20% were in North America, and 14% were in Arab Gulf countries. Whether the respondent had been resident in other countries since leaving Libya was not investigated.

Almost all respondents (72, 97%) obtained their first medical degree in Libyan universities; only two physicians obtained it elsewhere. Most respondents were working primarily as clinicians providing services (67 respondents, 91%), of whom 28 respondents (38%) were also active in clinical research. The profession of the remaining seven respondents (10%) was medical research not involving direct patient care.

Most of the respondents felt professionally well settled (53 respondents, 72%) or reasonably settled (18 respondents, 24%). However, they seemed to fare less well in terms of social integration, with only 16 (22%) stating that they were socially well integrated, and 34 (46%) and 21 (28%) saying that they were reasonably or partially integrated, respectively.

### Reasons for leaving Libya and staying abroad

The most important reason given for having gone abroad initially was the desire to further education and research (65 respondents ranked it first, 88%), while 9 respondents (12%) gave first rank to seeking a better income and living standard. However, the reasons given for deciding to say abroad showed a different pattern (Table 1): 37 respondents (50%) stated that the main reason was professional (further education and research), and 23 respondents (31%) gave economic factors as the main reason. Fourteen respondents (19%) stated that personal or family reasons were the main cause of their residence abroad.

**Table 1:** The first-ranked reason for staying abroad as ranked by 74 expatriate Libyan doctors.

Reason	Number	Percent
Educational: Pursue further education and research	37	50

Economic: Seek better income and life standard	23	31

Personal and other reasons:	14	19

• Poor working conditions in Libya, lack of job satisfaction, and more stability abroad	5	7

• Personal and family reasons	4	5

• Political reasons	2	3

• Religious reasons	2	3

• Better education for children	1	1

Data from survey of expatriate Libyan physicians, 2008, conducted by Benamer, Bredan and Bakoush.

For those who were in Europe, professional factors (education and research) were the most commonly given reasons for having settled abroad (31, 63%). By contrast, economic and social factors were the reasons given most frequently by those who settled in Arab countries (8, 80%) or in North America (11, 73%). Of those in Europe, 38.8% said they were not integrated or only partly integrated, compared to 20% of those in Arab countries and also 20% of those in North America.

Doctors who have settled abroad for professional and educational reasons were more active in biomedical research (23, 62%) compared to those staying for economic or social reasons (11, 31%).

### Returning to Libya

None of the participants ruled out the possibility of returning to Libya. About half of the respondents (54%) stated that they definitely or probably will return to Libya, and 46% described the likelihood of their return as possible or unlikely. The average length of stay abroad was shorter for those who were most likely to return compared to those who were less likely to return (12 and 16 years, respectively).

Most respondents (43, 58%) ranked reform of the Libyan health system as the most important reason that could motivate them to return to Libya (Table 2).

**Table 2:** The first-ranked reason that could motivate doctors to return to Libya (n = 74).

Reason	Number	Percent
Reform of the Libyan health services	43	58

Improvement in the prospects for research in the country	6	8

Social reasons	17	23

Other reasons:	8	11

• Overall improvement in the country's government system and infrastructure and more political freedom and transparency	7	10

• Changes in the circumstances in the destination country	1	1

Data from survey of expatriate Libyan physicians, 2008, conducted by Benamer, Bredan and Bakoush.

Most respondents said that if they were return to work in Libya, they would most likely work in a public hospital (29, 39%) or an academic institution (27, 36%). Only 11 respondents (15%) ranked work in the private sector as their first choice.

## Discussion

Our study shows that the most important reasons given by Libyan physicians for emigration are the pursuit of further education or research opportunities and the search for better income and lifestyles. These factors have been reported by others as reasons for emigration from poor to rich countries [11-13]. However, our findings are remarkable in that Libya is not a low-income country but is classified as an upper-middle income country [8]. To compensate for losing its doctors, Libya imports foreign doctors, who comprise 16% of all doctors working in the country [8].

Our results point to the attraction of career opportunities in destination countries with well established health infrastructures and academic institutions and advanced technology. The importance of these opportunities might even trump the factors of salary or lifestyle amenities already available in an upper-middle income country such as Libya.

There is general agreement among Libyan health care officials and Libyan doctors inside and outside Libya that the Libyan health care system requires considerable reform. Significantly, the results also indicate that such reform could be the strongest motivation for expatriate doctors to return home. Whether reform of health care delivery *per se *(e.g. increase in the number of hospitals or doctors) or the conditions of employment (e.g. increase in salary or modification of criteria for promotion) are more important in this respect requires further investigation. Gaining an understanding of what would motivate repatriation is as important as what causes migration, and such understanding would be useful in planning reforms.

Effective reform of the Libyan health care system is possible because the country enjoys a healthy economy, with an estimated per capita income of more than $7000 [8]. However, only 3.3% of the gross domestic product is spent on the health sector, compared to 4.3% in Algeria, 5.8% in Tunisia, 4.9% in Egypt, 9.2% in Sweden and 14.6% in USA [8]. To enhance career opportunities for Libyan doctors, we suggest increasing the share of health spending combined with serious steps to reform the health care system and increasing the remuneration of doctors (currently the senior physician earns about $35,000 a year, unadjusted to the cost of living). We propose that this will help reduce the emigration of Libyan doctors and motivate some expatriate Libyan physicians to return. Also, about 10% of the respondents suggested that overall improvement in the country's government and political system will help to induce doctors to return. The political reforms that started recently in the country could also help stem some of the brain drain from Libya.

Recruiting participants in the survey was made difficult by the absence of a database for expatriate Libyan doctors. We sought to recruit participants in a non-biased way from the database of the Libyan Journal of Medicine and members of the Libyan Doctors Society (a society for expatriate doctors). It might be argued that this led to recruitment only of doctors who are more "visible." But by asking the participants to help recruit others through their personal means we sought to reach others who were not "visible" through the society and the journal, and also to increase sample size.

Despite our efforts to maximise sample size, we were able to contact only about one-third of the reported expatriate Libyan physicians, and the response rate was disappointingly low. This limits the possibility of making generalisations and comparing different groups in the study, such as those residing in different countries. Our results should be viewed as preliminary data that give broad indications of the reasons for migration and the potential motivations for returning to Libya. Our results can be useful when planning studies of this type, both in terms of capitalising on its positive aspects and by avoiding its limitations. We support the suggestion made by Stilwell that data collection should be improved by establishing and maintaining a database on migration [14]. Such a database would be a valuable asset for future research on this phenomenon.

The relatively low response rate in this study might reflect a lack of interest among Libyan expatriate doctors in this issue. It might also reflect a belief among them that science journals would have no interest in this issue as it relates to a small country such as Libya, as stated by one respondent. Therefore, raising awareness of medical professionals about the significance of the migration issue is crucial for facilitating future studies and reducing the impact of this limitation.

We were somewhat surprised that only one of the 74 respondents was a woman, because in Libya women occupy a much larger share of this profession. We could not find officials figures, but the ratio of females to males is probably closer to at least 1:2. The 1:64 ratio we observed is difficult to account for, but we suspect that two social reasons could be involved. First, female doctors are probably less likely to travel abroad for postgraduate training, particularly if they are not married or their spouses do not approve. Second, female doctors in postgraduate training outside Libya are probably less likely to settle abroad than their male counterparts if they are unmarried or if their spouses do not agree. Moreover, some specialties, *e.g. *paediatrics, are staffed mostly by females whereas others, such as surgery, are staffed mostly by males. It is possible that those in female-dominated specialties are less likely to be sent abroad for post-graduate studies and specialisation.

The ethical issues that arise when implementing a questionnaire-based study include potential risks that participation might entail. In our study, all measures were taken to respect the participants' privacy, and they were and are assured of anonymity. Moreover, the participants are highly trained professionals who are familiar with these issues and can make an independent decision as to whether they want to participate in a study of this nature. Last but not least, criticisms of the performance of the Libyan health system have been discussed openly in conferences in Libya and employment conditions criticised.

Another limitation of this study is that it included only Libyan doctors working abroad. Including the views of Libyan doctors who returned and those who never left the country would be more comprehensive. Moreover, gathering the views of health officials, possibly by conducting semi-structured individual interviews and focus groups, would add another dimension to this type of work. It would also be interesting to explore the intentions of the newly qualified Libyan doctors and medical students to see whether a culture of medical migration is developing in Libya as has occurred in some other countries [3,6]. However, due to logistic difficulties we elected to restrict our study to only one group, hoping that our results would motivate greater participation and more comprehensive studies in the future.

The study was not designed to look at the effects of the migration of Libyan doctors. However, a report from the World Health Organization about the health system profile in Libya in 2007 states that the density of physicians in Libya decreased from 13.7 per 10,000 of the population in 1995 to 12.5 in 2006 [8]. By contrast, there are 22 physicians per 10,000 in Egypt, 23 in Jordan, 28 in USA and 33 in Sweden [8]. The report also stated that "Libya still finds itself lacking in specialists in a number of key areas such as anaesthesia, cardiology and radiology" [8]. The drain of doctors from Libya might be one of the factors contributing to the reduction in the ratio of doctors and specialists to the population.

## Conclusion

Taking in consideration all the limitations of this study, it is reasonable to conclude that reform of Libya's health system, including improvement of the conditions of practice and acceleration of investments in research and academic infrastructure, might motivate some of the physicians who moved abroad mainly for professional and economic reasons to return to their home country to practice medicine. The policymakers should take this point into consideration if they wish to manage this worrying phenomenon and avoid its consequences. Further studies looking at other issues related to the migration phenomenon, such as the negative impact on the country and whether an emigration culture is emerging, are needed to get more insight into the drain of doctors from Libya.

## Competing interests

The authors declare that they have no competing interests.

## Authors' contributions

HB conceived and designed the study, participated in data collection, analysis and interpretation, and drafted the manuscript. AB helped in designing the study, analysis and interpretation of data, and critical review of intellectual content. OB participated in design of the study, analysis and interpretation of data, and critical review of intellectual content. All authors read and approved the final manuscript.

## Supplementary Material

Additional file 1 **The study questionnaire**. The questionnaire used to collect data from the study participants. Click here for file
