# Supplementary material for: The Libyan doctors' brain drain: an exploratory study
Source: BMC Res Notes. 2009 Dec 8;2:242. doi: 10.1186/1756-0500-2-242 (PMC3225812; doi:10.1186/1756-0500-2-242)
Supplement: Additional file 1 — The study questionnaire. The questionnaire used to collect data from the study participants. [file 1756-0500-2-242-S1.DOC]

**Project: Libyan Doctors Brain Drain**

The goal of this project is to study some aspects of the brain drain of Libyan doctors, with the aim of shedding some light on the causes that initiate or help maintain this phenomenon.

We plan to collect the data by an anonymous questionnaire and publish the results in an international journal. We will keep the information you provide us in the strictest confidence and we will only use it to write the research paper.

We hope that you will assist us by filling out the following brief questionnaire. Please read each question carefully and be as accurate and honest as you can be. We thank you for your cooperation.

***Project’s Team***

*Hani Benamer, PhD, FRCP benamer@doctors.org.uk*

*Amin Bredan, PhD amin.bredan@dmbr.ugent.be*

*Omran Bakoush, MD, PhD Omran.Bakoush@med.lu.se*

**Libyan Doctors Brain Drain**

| **1. Gender** Male Female | **2. Age**       years | | | | | **3. Year you left Libya** | | |
| --- | --- | --- | --- | --- | --- | --- | --- | --- |
| **4. Place of qualification**  Libya Arab country Europe North America Other | | | | | | | | |
| **5. What was your original reason for going abroad? Please choose one or more answers.**  **If more than one, prioritise your choices as 1, 2, 3, etc.**  Educational: to further my education and research work  Economic: to seek better income and life standard  Personal: for family or other personal reasons  Any other reasons (specify) | | | | | | | | *select priority* |
|  |
| **6. What was your reason for deciding to stay abroad? Please choose one or more answers. If more than one, proritise your choices as 1, 2, 3, etc.**  Educational: to carry on with my education and research work  Economic: to seek better income and life standard  Personal: for family or other personal reasons  Any other reasons  (specify) | | | | | *select priority* | | **7. Country of current residence.**  Gulf country  Other Arab country  Europe  North America  Other | |
| **8. What is the best description of your current job? If more than one, prioritise your choices as 1, 2, 3, etc.**  Patient Care (clinical, service)  Medical research (academic)  Management (director, chief)  Other specify | | *select priority* | **9. How settled professionally are you in your current country of residence?**  Well settled  Reasonably settled  Only partially settled  Not settled | | | | **10. How integrated socially are you in your current country of residence?**  Well integrated  Reasonably integrated  Only partially integrated  Not integrated | |
| **11. What is the likelihood that you will return to practice medicine in Libya at any time in the future?**  Definitely Probably Possibly Unlikely Never | | | | | | | | |
| **12. What would induce you to return to practice medicine in Libya at any time in the future? Please choose one or more answers. If more than one, prioritise your choices as 1, 2, 3, etc.**  Significant reform of the Libyan health care system  Significant improvement in the prospects for research in Libya  Social reasons  Other reasons (specify)        Not returning under any circumstances | | | | | | | | *select*  *priority* |
|  |
| **13. If you decide to return to Libya permanently, what type of employment would you take up? Please check only ONE box.**  in a public hospital in an academic institute  in a private sector a non-medical job  other (specify) | | | |  | | | | |
|  | | | | |
|  | | | | |

Click-select inside check box*.* To type in text box     click word preceding it. Select priority from drop-down list.
